# The Role of Intraamygdaloid Oxytocin and D2 Dopamine Receptors in Reinforcement in the Valproate-Induced Autism Rat Model

**DOI:** 10.3390/biomedicines10092309

**Published:** 2022-09-16

**Authors:** Kristóf László, Dávid Vörös, Orsolya Kiss, Bettina Réka László, Tamás Ollmann, László Péczely, Kitti Mintál, Attila Tóth, Anita Kovács, Olga Zagoracz, Erika Kertes, Veronika Kállai, Beáta Berta, Zoltán Karádi, László Lénárd

**Affiliations:** 1 Medical School, Institute of Physiology, University of Pécs, Szigeti Str. 12, P.O. Box 99, 7602 Pécs, Hungary; 2 Neuropeptides, Cognition, Animal Models of Neuropsychiatric Disorders Research Group, Medical School, Institute of Physiology, University of Pécs, 7602 Pécs, Hungary; 3 Neuroscience Center, University of Pécs, 7602 Pécs, Hungary; 4 Learning in Biological and Artificial Systems Research Group, Medical School, Institute of Physiology, University of Pécs, 7602 Pécs, Hungary; 5 Cellular Bioimpedance Research Group, Szentágothai Research Center, University of Pécs, 7602 Pécs, Hungary; 6 Molecular Endocrinology and Neurophysiology Research Group, Szentágothai Center, University of Pécs, 7602 Pécs, Hungary

**Keywords:** autism, oxytocin, dopamine, amygdala, place preference, reinforcement, valproate

## Abstract

Background: autism spectrum disorder (ASD) is a neurodevelopmental disorder affecting around 1 out of 68 children and its incidence shows an increasing tendency. There is currently no effective treatment for ASD. In autism research, the valproate (VPA)-induced autism rodent model is widely accepted. Our previous results showed that intraamygdaloid oxytocin (OT) has anxiolytic effects on rats showing autistic signs under the VPA-induced autism model. Methods: rats were stereotaxically implanted with guide cannulae bilaterally and received intraamygdaloid microinjections. In the present study, we investigated the possible role of intraamygdaloid OT and D2 dopamine (DA) receptors on reinforcement using VPA-treated rats in a conditioned place preference test. OT and/or an OT receptor antagonist or a D2 DA antagonist were microinjected into the central nucleus of the amygdala (CeA). Results: valproate-treated rats receiving 10 ng OT spent significantly longer time in the treatment quadrant during the test session of the conditioned place preference test. Prior treatment with an OT receptor antagonist or with a D2 DA receptor antagonist blocked the positive reinforcing effects of OT. The OT receptor antagonist or D2 DA antagonist in themselves did not influence the time rats spent in the treatment quadrant. Conclusions: Our results show that OT has positive reinforcing effects under the VPA-induced autism rodent model and these effects are OT receptor-specific. Our data also suggest that the DAergic system plays a role in the positive reinforcing effects of OT because the D2 DA receptor antagonist can block these actions.

## 1. Introduction

The core symptoms of ASD include restrictive, repetitive, and stereotypic behavior, impairment of social interaction, communication disorders and cognitive rigidity [1]. There are significant gender differences in its incidence, with males being four times more likely to develop ASD than females [2]. The incidence of ASD has reached nearly 1.5% and a further significant increase is predicted [2]. Meanwhile, appropriate treatment for ASD is not yet available. Consequently, ASD places an economic burden on society and a future increase in ASD costs is expected, while the total-base costs were estimated to be USD 223 billion/year in 2020, USD 589 billion/year in 2030, USD 1.36 trillion/year in 2040, and USD 5.54 trillion/year by 2060, considering only the USA [3].

ASD is a neurodevelopmental disorder, so the first symptoms appear in early childhood and the diagnosis is based on these behaviors [2,4]. ASD is known to co-occur with some neuropsychiatric disorders, such as schizophrenia, epilepsy, anxiety, sleep disturbances, fragile X syndrome and mental retardation [4]. Numerous risk factors can lead to the development of ASD, inter alia, genetics, environmental pollution, high parental age, low birth weight, intrauterine infection and drug exposure (i.e., valproate exposure) [5,6].

VPA is a widely used, broad-spectrum antiepileptic drug. VPA administration is not only indicated for epilepsy but also for migraine, dementia, bipolar disease and neuropathic pain [7]. However, VPA is a teratogenic medicine and significantly increases the risk of ASD development [8]. Based on the aforementioned information, VPA is a good candidate for inducing autistic-like behavior in rodents. Studies show that the optimal method of administering VPA to induce autistic-like behavior is if the dams receive it at a dose of 500 mg/bwkg intraperitoneally on the 12.5th day of gestation [9]. Due to the intrauterine VPA exposure, the offspring animals show autistic-like behavior such as impaired social interaction, restrictive, repetitive behavior, and anxiety [10]. Rodents exposed to intrauterine VPA show significantly less ultrasonic vocalization when separated from their dam and siblings [11,12,13]. Based on the aforementioned details, prenatal exposure to VPA is validated as a drug-induced animal model of ASD. Our previous study showed that intraamygdaloid OT has anxiolytic effects in VPA-treated rats [14] and our previous data also indicate that intraamygdaloid OT reduces social impairments in rats showing autistic signs (under publication).

Dysfunction of the OTergic system has a potential role in the pathogenesis of neuropsychiatric diseases associated with social impairment, such as ASD, social phobia and schizophrenia [15]. OT is mainly produced by the paraventricular, supraoptic and accessory nuclei of the hypothalamus and it is released into numerous brain regions including the central nucleus of the amygdala (CeA), acting as a neurotransmitter/neuromodulator [16,17,18]. OT is also released into the blood stream from the posterior pituitary gland and has an important role in coordinating birth, lactation and maternal care. OT is well-known for its prosocial effects and it is involved in grooming, parental behavior, bonding, sexual behavior, social memory, trust and social recognition [18,19]. The fact that it also plays a role in the regulation of pain perception, food, and fluid intake shows the versatile effects of OT [20,21]. We have previously shown that OT microinjected into the CeA of neurotypical rats has positive reinforcing effects and that these effects are OT receptor-specific [22]. Our previous data suggested a role for the DAergic system in the rewarding effects of OT [23]. It is known that the CeA receives dopaminergic fibers from the mesolimbic dopaminergic system (MLDS). The amygdala (AMY) is involved in the regulation of anxiety, fear-related behavior, sexual behavior, food intake, reinforcement and motivation as part of the limbic system. OTergic fibers innervate the CeA, which has been shown to be rich in OT receptors [16,17,24].

General hypoactivation of the reward system has been implicated in ASD [25,26,27,28]. Earlier we showed that OT has rewarding effects when microinjected into neurotypical rats’ CeA [22] and our data also suggest a role for the MLDS in these effects [23]. It is also a question whether OT also has positive reinforcing effects in rats showing autistic signs, even though the reward system is presumably hypoactive in these animals. It is also unclear whether manipulation of D2 DA receptor activity might play a role in these effects. The CeA is innervated by the MLDS and receives OTergic fibers as well. Furthermore, the CeA is relatively rich in OT and D2 DA receptors [29,30]. Therefore, the goal of the present study was to investigate the possible effects of OT and the D2 DA receptor antagonist Sulpiride in the rat CeA on reinforcement in the place preference test under the VPA-induced autism rodent model.

## 2. Materials and Methods

### 2.1. Subjects

The VPA-induced autism rat model was employed. Female rats in estrus phase (determined from vaginal smears) were mated overnight. Gestational day 1 was assigned as the morning when spermatozoa were found. On the 12.5th day of gestation, dams received a single intraperitoneal injection of 500 mg/bwkg valproate (Sigma-Aldrich Kft., Budapest, Hungary; P4543) dissolved in saline at a concentration of 250 mg/mL, and control females were injected with physiological saline. Dams were allowed to raise their own litters in their individual homecages. In our experiments, 79 adult male (neurotypical and autistic signs showing) Wistar rats weighing 270–290 g at the time of surgery were housed individually and cared for in accordance with institutional (BA02/2000-8/2012, BA02/2000-64/2017, BA02/2000-04/2021), national (Hungarian Government Decree, 40/2013 (II. 14.)), and international standards (European Community Council Directive, 86/609/EEC, 1986, 2010). The autistic-like behavior of male offspring was determined at the ages of 4 and 8 weeks by means of a social interaction test and an open field test. The VPA-exposed rats which showed less social interaction and more repetitive behavior than control rats were chosen. Animals were kept in a temperature- and light-controlled room (22 ± 2 °C; 12:12 h light–dark cycle with lights on from 6:00 a.m.). Standard laboratory food pellets (CRLT/N standard rodent food pellet, Charles River Kft, Budapest, Hungary) and tap water were available ad libitum. All behavioral tests were performed during the rats’ daylight period, between 08:00 a.m. and 4:00 p.m.

### 2.2. Surgery

Surgery was carried out under general anesthesia maintained intraperitoneally by ketamine supplemented with diazepam (Calypsol and Seduxen, Richter Gedeon, Budapest, Hungary; ketamine: 80 mg/kg body weight; diazepam: 20 mg/kg body weight). Twenty-two-gauge stainless steel guide cannulae were stereotaxically and bilaterally implanted, directed toward and 1 mm above the dorsal border of the CeA (coordinates relative to the bregma: AP: −2.3 mm, ML: ±4.1 mm, DV: −6.5 mm), according to the rats’ stereotaxic atlas [31]. To fix the cannulae to the skull, three stainless steel screws and dental acrylic were used. When not being used for injection, the guide cannulae were closed up with 27-gauge stainless steel obturators. A minimum 6 days of postoperative recovery were allowed before starting the experiments. During this time, rats were handled daily.

### 2.3. Drugs and Injection Procedure

OT obtained from Sigma (Sigma-Aldrich Co., St. Louis, MO, USA, O6379) was microinjected bilaterally. The applied dose was 10 ng (9.93 pmol) in a 0.4 µL volume per side. OT was dissolved in 0.15 M sterile saline solution containing 0.01 M Na-acetate and 0.01 M phosphate-buffered saline (PBS, pH 7.4). Control animals received this solution bilaterally as a vehicle at a volume equal to that used for OT injections. The OT receptor antagonist L-2540 [Sigma-Aldrich Co., L-368–899, 20 ng (16.92 pmol)/0.4 µL)] was diluted in 0.15 M saline solution containing 0.01 M Na-acetate and 0.01 M phosphate-buffered saline (PBS, pH 7.4). The D2 DA antagonist Sulpiride [Sigma-Aldrich Co., S7771, 4 µg/0.4 µL] was diluted in 0.15 M saline solution containing 0.01 M Na-acetate and 0.01 M phosphate-buffered saline (PBS, pH 7.4). Altogether, 10 groups of animals were involved in the conditioned place preference (CPP) tests. There were five groups in the first CPP experiment. To be specific, the five groups were the Control group (neurotypical/sham rats, which received bilateral vehicle microinjections), the VPA (intrauterine valproate-treated animals showing autistic-like behavior, which received bilateral vehicle microinjections), and VPA + 10 ng OT groups (intrauterine valproate-treated animals showing autistic-like behavior, which received a bilateral intraamygdaloid treatment of 10 ng OT), the VPA + ANT + OT group (intrauterine valproate-treated animals showing autistic-like behavior, which received 10 ng OT and were pretreated with 20 ng OT receptor antagonist 15 min earlier), and the VPA + ANT group (intrauterine valproate-treated animals showing autistic-like behavior, which received 20 ng OT receptor antagonist). There were also five groups (new animals) in the second CPP test. Specifically, the groups were the Control group (neurotypical/sham rats, which received bilateral vehicle microinjections), the VPA (intrauterine valproate-treated animals showing autistic-like behavior, which received bilateral vehicle microinjections) and VPA + 10 ng OT groups (intrauterine valproate-treated animals showing autistic-like behavior, which received a bilateral intraamygdaloid treatment of 10 ng OT), a VPA + D2 ANT + OT group (intrauterine valproate-treated animals showing autistic-like behavior, which received 10 ng OT and were pretreated with 4 µg D2 DA receptor antagonist Sulpiride), and a VPA + D2 ANT group (intrauterine valproate-treated animals showing autistic-like behavior, which received 4 µg D2 DA receptor antagonist Sulpiride). To avoid the litter effect, only one rat was chosen from each litter of a given group. Solutions were kept at +4 °C before application. In this article, all doses are reported as dose per side values. Drugs or vehicles were bilaterally microinjected through a 30-gauge stainless steel injection tube extending 1 mm below the tips of the implanted guide cannulae. The injection cannula was attached via polyethylene tubing (PE-10) to a 10 µL Hamilton microsyringe (Hamilton Co., Bonaduz, Switzerland). All injections were delivered by a syringe pump (Cole Parmer, IITC, Life Sci. Instruments, Vernon Hills, IL, USA) in a 0.4 µL volume over a 60 s interval. After injection, cannulae were left in place for an additional 60 s to allow diffusion into the surrounding tissue. During the injections, rats were gently held in hand.

### 2.4. Conditioned Place Preference (CPP) Test

One way to measure the positive reinforcing effects of a drug is the CPP test [32]. The apparatus we used consisted of a circular open field with a diameter of 85 cm and a height of 40 cm, which was divided into four equal-sized quadrants by black lines. External visual cues outside the apparatus helped the rats’ spatial orientation [33]. The room was dimly lit at 40 lux. The place preference procedure consisted of one Habituation (day 1), two Conditioning (day 2–3) and one Test (day 4) trials; each lasted 900 s (15 min). The apparatus was cleaned and dried after each animal used it. All trainings and tests were conducted in an isolated experimental room. On day 1 (Habituation), animals were placed into the arena and had free access to all parts of the apparatus for 900 s. The time the animals spent in each of the four quadrants was measured. During Conditioning trials (day 2–3), the animals received the drug injections (see “Drugs and Injection Procedure”) and subsequently the rats were restricted to the treatment quadrant for 15 min by means of a transparent plexiglass barrier. The treatment quadrant was determined to be one of the four quadrants in which the animal had spent neither the longest nor the shortest time during habituation [33,34]. Due to the homogenous environment, there was no initial quadrant preference. On the fourth day (Test trial), animals had free access to all parts of the apparatus. The time that the rats had spent in each of the four quadrants was measured again. The animals’ behavior was recorded with a video camera. The data were stored and motion analysis was conducted by means of the EthoVision Basic software (Noldus Information Technology B.V., Wageningen, The Netherlands). The number of entries into the four quadrants was also recorded during the Habituation and Test trials as a measure of gross locomotor activity.

### 2.5. Histology

When the experiments ended, rats received an overdose of Calypsol and Seduxen blended in a ratio of 4:1 and were transcardially perfused with isotonic saline followed by 10% formalin solution. After 1 week of postfixation, the brains were frozen, and for histological review, 40 µm serial sections stained with Cresyl-violet were examined. Injection sites were reconstructed according to the stereotaxic atlas of the rat brain [31]. Only data from rats with correctly placed cannulae were analyzed.

### 2.6. Statistical Analysis

The data are presented as mean ± standard error of the mean (S.E.M.). Mixed ANOVAs followed by Tukey’s post hoc analysis were employed. Statistical significance was established at *p* < 0.05 (IBM SPSS Statistics 26, Powell, OH, USA).

## 3. Results

### 3.1. Histology

**Figure 1:**
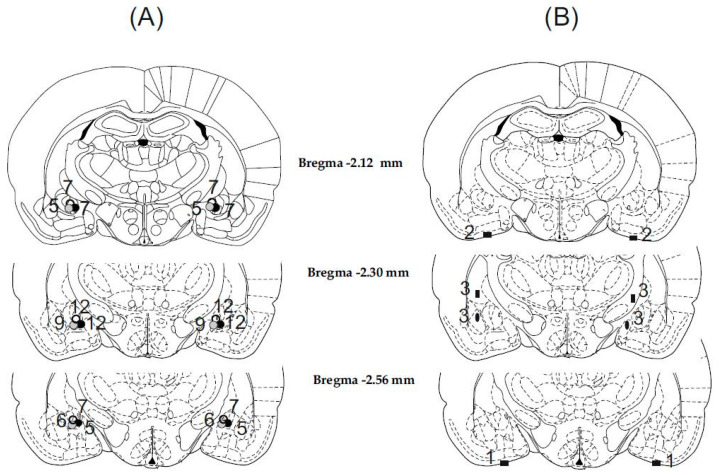
Illustration of reconstructed injection sites. Correct bilateral injection placements are indicated by closed gray and black circles in the CeA in panel (**A**) (*n* = 70). Incorrect injection placements are indicated in panel (**B**) (*n* = 9). Brain structure diagrams of coronal sections are adapted from the stereotaxic atlas of Paxinos and Watson. The numbers refer to anterior–posterior distances from the bregma in mm. Identical symbols in panel (**B**) indicate coherent injection sites of bilateral injections. Numbers above marked sites in panels (**A**,**B**) indicate numbers of animals.

Histological examination showed that in 70 cases out of 79 animals, the cannulae were precisely and symmetrically located in the target area (the CeA). The tracks of the cannulae and tip positions were determined by examining for evidence of debris and moderate glial proliferation. A schematic illustration of cannula placements is shown in Figure 1.

The nine rats with misplaced injection sites were excluded from subsequent analysis (Figure 1B). Among these rats, in three cases, the cannula tips were symmetrically inserted into the liquor space at the basis of the brain. In three cases, the cannula tips were located laterally or medially and 1 mm above the AMY, thus injections were made in the caudate-putamen on one side and in the internal capsule on the other side. In three cases, the cannula tips were placed laterally or medially to the target area, therefore injections were made in the lateral and basolateral AMY or in the medial AMY nucleus. Behavioral data concerning these incorrect and diverse placements were not enough to draw far-reaching conclusions.

### 3.2. Conditioned Place Preference Test

**Figure 2:**
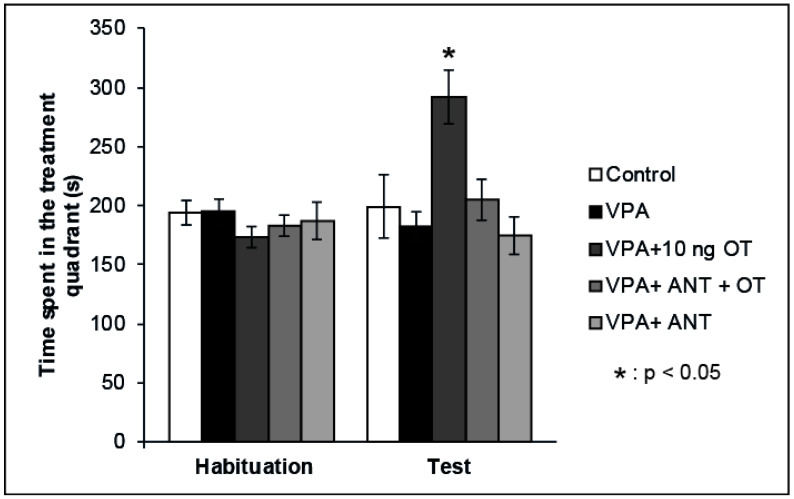
Effects of OT and the OT receptor antagonist injected into the CeA of autistic rats on the conditioned place preference (CPP) test. Columns represent mean time spent in the treatment quadrant (±S.E.M.) during Habituation and Test sessions. Control: neurotypical/sham male Wistar rats receiving intraamygdaloid PBS (*n* = 6); VPA: intrauterine VPA-treated rats showing autistic-like behavior (*n* = 7); VPA + 10 ng OT: animals showing autistic-like behavior, microinjected with 10 ng OT (*n* = 7); VPA + ANT + OT: animals with autistic-like behavior, microinjected with 20 ng OT receptor antagonist and 10 ng OT (*n* = 7); VPA + ANT: animals with autistic-like behavior, microinjected with 20 ng OT receptor antagonist (*n* = 7); * *p* < 0.05; for more explanation, see the text.

Our previous data showed that OT has positive reinforcing properties when microinjected into the rat CeA [22]. Therefore, we examined whether the positive reinforcing effects of OT can be established in rats showing autistic signs. The effects of intraamygdaloid OT and/or OT receptor antagonist on the time spent in the treatment quadrant during the CPP test are shown in Figure 2. Mixed ANOVA analysis revealed a significant effect of trials [F(1,34) = 5.746, *p* < 0.05] and there was a significant effect of treatment [F(3,34) = 4.883, *p* < 0.05], along with a significant interaction between treatment and trials [F(3,32) = 6.189, *p* < 0.01]. The VPA + 10 ng OT (*n* = 7) treatment increased the time animals spent in the treatment quadrant during the Test session compared to the time spent by the Control group (*n* = 6, *p* < 0.05) and compared to that spent during Habituation (*p* < 0.05). When the OT receptor antagonist (*n* = 7) was administered prior to the 10 ng OT treatment, it blocked the positive reinforcing effect (*p* < 0.05). OT receptor antagonist administered alone (*n* = 7) failed to have any effect on place preference behavior on its own: the time spent in the treatment quadrant during the Test session did not differ from that spent by Control animals (*n* = 6, N.S.), but significantly differed from that spent by the 10 ng OT-treated group (*n* = 7, *p* < 0.05). To avoid the litter effect, only one rat was chosen from each litter of a given group.

The gross locomotor activity (distance moved in cm) of the rats was measured during the entire experiment. The subjects were in a drug-free state during the Habituation and Test trials and there were no significant differences among the different groups, and the Habituation vs. Test trials did not show significant differences as far as the covered distance is concerned (Table 1). During the Conditioning trials, when the animals received the drug injections and right after the rats were restricted to a treatment quadrant, there were no statistical differences regarding locomotor activities (Table 1), rearing and freezing (data are not shown). Animals covered a shorter distance during the Conditioning trials than in the Habituation and Test trials because they were restricted to a treatment quadrant (Table 1).

**Figure 3:**
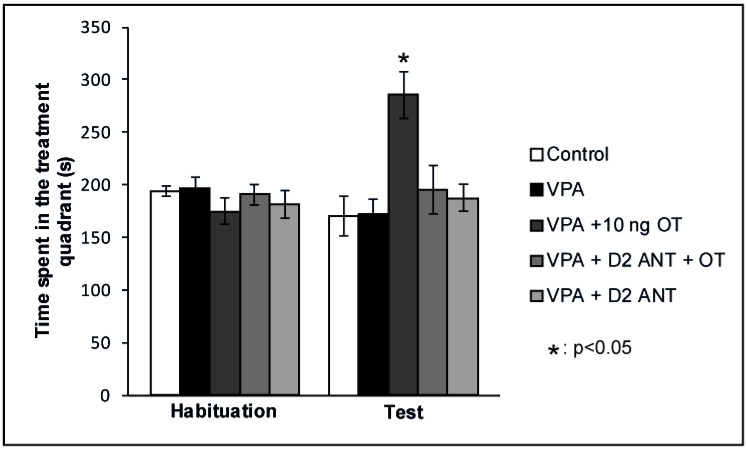
Effects of D2 DA antagonist pretreatment in the CeA of autistic rats on the conditioned place preference (CPP) test. Columns represent mean time spent in the treatment quadrant (±S.E.M.) during Habituation and Test sessions. Control: neurotypical/sham male Wistar rats receiving intraamygdaloid PBS (*n* = 8); VPA: intrauterine VPA-treated rats showing autistic-like behavior (*n* = 7); VPA + 10 ng OT: animals showing autistic-like behavior, microinjected with 10 ng OT (*n* = 7); VPA + D2 ANT + OT: animals with autistic-like behavior, microinjected with 10 ng OT and pretreated with 4 µg Sulpiride (*n* = 7); VPA + D2 ANT: animals with autistic-like behavior, treated with 4 µg Sulpiride (*n* = 7); * *p* < 0.05, for more explanations, see the text.

According to our hypothesis, the positive reinforcing effects of OT on rats showing autistic signs may be mediated via the modulation of the MLDS. Therefore, we examined whether the positive reinforcing effects of OT can be attenuated by blocking the D2 DA receptors in the valproate-induced autism model. The effects of intraamygdaloid D2 DA antagonist pretreatment on the time spent in the treatment quadrant during the CPP test are shown in Figure 3. Mixed ANOVA analysis revealed a significant effect of trials [F(1,35) = 4.648, *p* < 0.05] and there was a significant effect of treatment [F(3,35) = 3.441, *p* < 0.05], along with a significant interaction between treatment and trials [F(3,35) = 6.536, *p* < 0.01]. The VPA + 10 ng OT (*n* = 7) treatment increased the time animals spent in the treatment quadrant during the Test session compared to the time spent by the Control group (*n* = 8, *p* < 0.05) and compared to that spent during Habituation (*p* < 0.05). When the D2 DA antagonist Sulpiride was administered prior to the 10 ng OT treatment (*n* = 7), it blocked the positive reinforcing effect (*p* < 0.05). Sulpiride administered alone (*n* = 7) failed to have any effect on place preference behavior on its own: time spent in the treatment quadrant during the Test session did not differ from that spent by Control animals (*n* = 8), but significantly differed from the time spent by the 10 ng OT-treated group (*n* = 7, *p* < 0.05). To avoid the litter effect, only one rat was chosen from each litter of a given group.

The gross locomotor activity was measured this time as well; see details above. The subjects were in a drug-free state during the Habituation and Test trials and there were no significant differences among the different groups, and the Habituation vs. Test trials did not show significant differences as far as the covered distance is concerned (Table 2). During the Conditioning trials, when the animals received the drug injections and right after rats were restricted to a treatment quadrant, there were no statistical differences regarding locomotor activities (Table 2), rearing and freezing (data are not shown).

## 4. Discussion

Our previous results showed that OT has positive reinforcing effects on neurotypical rats [22] and these effects could be blocked by a D2 DA receptor antagonist [23]. In line with our previous data, the present results suggest that the rewarding effects of intraamygdaloid OT do exist in the valproate-induced autism rodent model as well. OT seems to have a strong reinforcing effect in the rat CeA, even though it has been reported that the reward system is hypoactive in autism [25,26,27,28].

Human data suggest that intranasal OT enhances social reinforcement learning in ASD via stimulating the motivational system [35]. Chemogenetic modulation of OT neurons has been shown to lead to social motivation [36]. Furthermore, in humans, OT has been reported to enhance socially reinforced learning that depends on AMY function [37]. The study of Gamer et al. also shows that OT increases AMY activity in response to pleasant stimuli but decreases AMY activity in response to unpleasant social stimuli [38].

In line with the aforementioned studies, OT has been shown to have positive reinforcing effects [22,39,40,41,42,43,44]. Specifically, OT receptor activity of the ventral tegmental area is crucial for the reinforcing properties of social interactions [39]. Moreover, OT has motivation-facilitating properties in conditioned place preference tests when administered peripherally [42]. In line with previous research, OT has been shown to induce conditioned place preference in female rats when microinjected into the lateral ventricle [41]. However, it has also been indicated that intranasal OT administration does not elicit place preference in female mice, but it leads to conditioned social place preference [45,46]. Development of place preference requires memory formation and motivation, but it is difficult to distinguish between motivation-related and memory aspects of CPP. However, the test trial depends on whether animals remember the treatment quadrant, which is a memory-related condition [47]. It is well known that the DAergic system is involved in reinforcement, as well as in learning and memory processes [48,49]. Can it be assumed that there is a connection between ASD and DAergic system dysfunction? Hypotheses suggest a role for DAergic dysfunction in autistic-like behavior [28,50]. While dysfunction of the MLDS might lead to social impairments, the dysfunction of the nigrostriatal DAergic system might result in stereotypic behavior [28,50]. The MLDS plays a crucial role in motivation and reinforcement. Consequently, dysfunction of this system may lead to altered motivation and reduced reward. This raises the possibility that autistic individuals do not find social interactions rewarding and have reduced motivation to seek social interaction [28]. This hypothesis is also supported by the social motivation theory. According to this theory, patients with ASD have reduced social motivation, which impacts social cognition and consequently leads to social impairments [28,51]. Further studies support the possible role of DAergic dysfunction in ASD as well. Specifically, reduced DA release and hypoactivation of the reward system have been reported in ASD, affecting not only social but also non-social reward-related behavior [25,26,27]. An association has been demonstrated between the OT receptor gene and the mesolimbic response to rewards, implying a facilitating role of OT in MLDS activity [52]. Indeed, an interaction exists between oxytocinergic and DAergic systems [53,54,55]. Co-localization of D2 DA receptors and OT receptors has been demonstrated in the CeA and in the striatum as well [30,56,57]. DAergic neurons of the VTA express OT receptors, and axons project to limbic structures [58]. Furthermore, OT has been demonstrated to modify DA release from the MLDS in mice [58]. Existence of OT receptor–D2 DA receptor heterocomplexes have been shown in the CeA and OT function can be enhanced by a D2 DA receptor agonist [59]. Facilitatory oxytocin–D2 DA receptor–receptor interactions have been shown to lead to an enhancement in the signaling of the oxytocin–D2 DA heteroreceptor complexes. The above might be part of the molecular mechanism for OT-induced changes in emotional and social behavior [59,60,61]. Furthermore, de la Mora et al. have shown that activation of the oxytocin receptor protomer can increase D2 DA protomer signaling in the oxytocin–D2 DA heteroreceptor complex via a facilitatory allosteric receptor–receptor interaction [59]. In line with these data, our data may allow us to conclude that a D2 DA antagonist could block the positive reinforcing effects of OT via inhibiting the OT receptor–D2 DA receptor heterocomplexes in the CeA. However, further studies are necessary to support this hypothesis and to clarify the possible role of the MLDS in our findings.

The VPA-induced autism rat model was used in our experiments. In light of our results, it may seem controversial that VPA has been shown to inhibit morphine-induced CPP [62], meanwhile in our experiment, intraamygdaloid OT administration resulted in place preference in VPA-treated rats. It is important to note that the route, site and dose of VPA injection, as well as the time between VPA administration and testing in that study were completely different from ours. In our experiments, dams were administered a single intraperitoneal 500 mg/bwkg VPA injection on the 12.5th day of pregnancy and offspring were tested about 12–14 weeks later. On the other hand, Ping Mu et al. applied a chronic VPA treatment intracerebroventricularly to fully grown animals, at a dose of 500 µg/rat, 10 min before morphine injection, which was followed by CPP testing [62]. However, Spyraki et al. have shown that VPA does not influence diazepam-induced place preference, but in this experiment, adult animals received VPA during testing as well [63].

One of the limitations of our study is that only male rats were examined. ASD is known to affect far more males than females [2]. On the other hand, the estrus cycle affects the behavior of female rats and it is very likely that the behavior of male rats would also be affected by the presence of female rats via pheromones [64].

There is currently no cure for autism, but in some respects, behavioral therapies based on reward-based reinforcement learning seem effective [65]. Studies also indicate the potential role of OT in ameliorating autistic symptoms and OT has been reported to play a role in social reward mechanisms [66,67,68]. Our present findings suggest that intraamygdaloid OT is involved in non-social positive reinforcing mechanisms under the VPA-induced autism rat model. Taken together, the aforementioned data raise the possibility that the combination of behavioral therapy with OT therapy can be worthwhile. However, further preclinical and clinical studies are necessary for a better understanding of ASD, for which our findings may provide some basis.

## Figures and Tables

**Table 1:** Distance moved during the CPP trial (±S.E.M.) Control: neurotypical/sham male Wistar rats receiving intraamygdaloid PBS (*n* = 6); VPA: intrauterine VPA-treated rats showing autistic-like behavior (*n* = 7); VPA + 10 ng OT: animals showing autistic-like behavior, microinjected with 10 ng OT (*n* = 7); VPA + ANT + OT: animals with autistic-like behavior, microinjected with 20 ng OT receptor antagonist and 10 ng OT (*n* = 7); VPA + ANT: animals with autistic-like behavior, microinjected with 20 ng OT receptor antagonist (*n* = 7). For more explanation, see the text.

Distance Covered (cm/15 min) (Avg ± SEM)	Habituation	Avg. of Conditioning Trials	Test
control (*n* = 6)	6572.32 ± 512.88	3912.52 ± 233.18	5963.25 ± 501.05
VPA (*n* = 7)	6464.11 ± 408.92	3475.56 ± 271.25	6075.21 ± 475.13
VPA + 10 ng OT (*n* = 7)	6213.33 ± 476.35	3712.45 ± 265.24	5888.51 ± 456.32
VPA + ANT + OT (*n* = 7)	6632.15 ± 555.41	3795.02 ± 279.66	5893.21 ± 515.00
VPA + ANT (*n* = 7)	6348.99 ± 423.85	3275.92 ± 253.88	5760.85 ± 490.14

**Table 2:** Distance moved during the CPP trials (±S.E.M.) Control: neurotypical/sham male Wistar rats receiving intraamygdaloid PBS (*n* = 8); VPA: intrauterine VPA-treated rats showing autistic-like behavior (*n* = 7); VPA + 10 ng OT: animals showing autistic-like behavior, microinjected with 10 ng OT (*n* = 7); VPA + D2 ANT + OT: animals with autistic-like behavior, animals microinjected with 10 ng OT and pretreated with 4 µg Sulpiride (*n* = 7); VPA + D2 ANT: animals with autistic-like behavior, treated with 4 µg Sulpiride (*n* = 7). For more explanation, see the text.

Distance Covered (cm/15 min) (Avg ± SEM)	Habituation	Avg. of Conditioning Trials	Test
control (*n* = 8)	6272.82 ± 502.11	4022.12 ± 283.19	5963.25 ± 501.05
VPA (*n* = 7)	6363.81 ± 468.52	3576.51 ± 291.13	5875.01 ± 485.26
VPA + 10 ng OT (*n* = 7)	6014.75 ± 456.99	3612.35 ± 285.87	5766.53 ± 446.38
VPA + D2 ANT + OT (*n* = 7)	6402.10 ± 535.84	3595.82 ± 299.16	5793.33 ± 495.12
VPA + D2 ANT (*n* = 7)	6248.34 ± 433.18	3375.99 ± 263.15	5569.68 ± 458.21

## Data Availability

https://drive.google.com/drive/folders/16dwVfadXB23lR28ifAgqRuhqVNHdhfwA?usp=sharing (accessed on3 February 2022).

## References

[1] American Psychiatric Association (2013). Diagnostic and Statistical Manual of Mental Disorders.

[2] Sharma S.R., Gonda X., Tarazi F.I. (2018). Autism Spectrum Disorder: Classification, diagnosis and therapy. Pharmacol. Ther..

[3] Blaxill M., Rogers T., Nevison C. (2022). Autism Tsunami: The Impact of Rising Prevalence on the Societal Cost of Autism in the United States. J. Autism. Dev. Disord..

[4] Karande S. (2006). Autism: A review for family physicians. Indian J. Med. Sci..

[5] Herrero M.J., Velmeshev D., Hernandez-Pineda D., Sethi S., Sorrells S., Banerjee P., Sullivan C., Gupta A.R., Kriegstein A.R., Corbin J.G. (2020). Identification of amygdala-expressed genes associated with autism spectrum disorder. Mol. Autism.

[6] Landrigan P.J. (2010). What causes autism? Exploring the environmental contribution. Curr. Opin. Pediatr..

[7] Perucca E. (2002). Pharmacological and therapeutic properties of valproate: A summary after 35 years of clinical experience. Cns Drugs.

[8] Wieck A., Jones S. (2018). Dangers of valproate in pregnancy. BMJ-Br. Med. J..

[9] Kim K.C., Kim P., Go H.S., Choi C.S., Yang S.I., Cheong J.H., Shin C.Y., Ko K.H. (2011). The critical period of valproate exposure to induce autistic symptoms in Sprague-Dawley rats. Toxicol. Lett..

[10] Tartaglionea A.M., Schiavi S., Calamandreiam G., Trezzam V. (2018). Prenatal valproate in rodents as a tool to understand the neural underpinnings of social dysfunctions in autism spectrum disorder. Neuropharmacology.

[11] Gandal M.J., Edgar J.C., Ehrlichman R.S., Mehta M., Roberts T.P.L., Siegel S.J. (2010). Validating gamma Oscillations and Delayed Auditory Responses as Translational Biomarkers of Autism. Biol. Psychiatry.

[12] Melancia F., Schiavi S., Servadio M., Cartocci V., Campolongo P., Palmery M., Pallottini V., Trezza V. (2018). Sex-specific autistic endophenotypes induced by prenatal exposure to valproic acid involve anandamide signalling. Br. J. Pharmacol..

[13] Moldrich R.X., Leanage G., She D., Dolan-Evans E., Nelson M., Reza N., Reutens D.C. (2013). Inhibition of histone deacetylase in utero causes sociability deficits in postnatal mice. Behav. Brain Res..

[14] Laszlo K., Kiss O., Voros D., Mintal K., Ollmann T., Peczely L., Kovacs A., Zagoracz O., Kertes E., Kallai V. (2022). Intraamygdaloid Oxytocin Reduces Anxiety in the Valproate-Induced Autism Rat Model. Biomedicines.

[15] Kanat M., Heinrichs M., Domes G. (2014). Oxytocin and the social brain: Neural mechanisms and perspectives in human research. Brain Res..

[16] Knobloch H.S., Charlet A., Hoffmann L.C., Eliava M., Khrulev S., Cetin A.H., Osten P., Schwarz K.M., Seeburg P.H., Stoop R. (2012). Evoked axonal oxytocin release in the central amygdala attenuates fear response. Neuron.

[17] Voorn P., Buijs R.M. (1983). An Immuno-Electronmicroscopical Study Comparing Vasopressin, Oxytocin, Substance-P and Enkephalin Containing Nerve-Terminals in the Nucleus of the Solitary Tract of the Rat. Brain Res..

[18] Grinevich V., Knobloch-Bollmann H.S., Eliava M., Busnelli M., Chini B. (2016). Assembling the Puzzle: Pathways of Oxytocin Signaling in the Brain. Biol. Psychiatry.

[19] Lee H.J., Macbeth A.H., Pagani J.H., Young W.S. (2009). Oxytocin: The great facilitator of life. Prog. Neurobiol..

[20] Verty A.N.A., McFarlane J.R., McGregor I.S., Mallet P.E. (2004). Evidence for an interaction between CB1 cannabinoid and oxytocin receptors in food and water intake. Neuropharmacology.

[21] Yang J., Yang Y., Chen J.M., Liu W.Y., Wang C.H., Lin B.C. (2007). Effect of oxytocin on acupuncture analgesia in the rat. Neuropeptides.

[22] Laszlo K., Kovacs A., Zagoracz O., Ollmann T., Peczely L., Kertes E., Lacy D.G., Lenard L. (2016). Positive reinforcing effect of oxytocin microinjection in the rat central nucleus of amygdala. Behav. Brain Res..

[23] Laszlo K., Peczely L., Geczi F., Kovacs A., Zagoracz O., Ollmann T., Kertes E., Kallai V., Laszlo B., Berta B. (2020). The role of D2 dopamine receptors in oxytocin induced place preference and anxiolytic effect. Horm. Behav..

[24] Condeslara M., Veinante P., Rabai M., Freundmercier M.J. (1994). Correlation between Oxytocin Neuronal Sensitivity and Oxytocin-Binding Sites in the Amygdala of he Rat—Electrophysiological and Histoautoradiographic Study. Brain Res..

[25] Dichter G., Adolphs R. (2012). Reward processing in autism: A thematic series. J. Neurodev. Disord..

[26] Dichter G.S., Felder J.N., Green S.R., Rittenberg A.M., Sasson N.J., Bodfish J.W. (2012). Reward circuitry function in autism spectrum disorders. Soc. Cogn. Affect. Neurosci..

[27] Ernst M., Zametkin A.J., Matochik J.A., Pascualvaca D., Cohen R.M. (1997). Low medial prefrontal dopaminergic activity in autistic children. Lancet.

[28] Paval D. (2017). A Dopamine Hypothesis of Autism Spectrum Disorder. Dev. Neurosci..

[29] de la Mora M.P., Gallegos-Cari A., Crespo-Ramirez M., Marcellino D., Hansson A.C., Fuxe K. (2012). Distribution of Dopamine D-2-Like Receptors in the Rat Amygdala and Their Role in the Modulation of Unconditioned Fear and Anxiety. Neuroscience.

[30] Gimpl G., Fahrenholz F. (2001). The oxytocin receptor system: Structure, function, and regulation. Physiol. Rev..

[31] Paxinos G., Watson C. (1986). The Rat Brain in Stereotaxic Coordinates.

[32] Tzschentke T.M. (1998). Measuring reward with the conditioned place preference paradigm: A comprehensive review of drug effects, recent progress and new issues. Prog. Neurobiol..

[33] Hasenohrl R.U., Oitzl M.S., Huston J.P. (1989). Conditioned place preference in the corral—A procedure for measuring reinforcing properties of drugs. J. Neurosci. Methods.

[34] Zimmermann P., Privou C., Huston J.P. (1999). Differential sensitivity of the caudal and rostral nucleus accumbens to the rewarding effects of a H1-histaminergic receptor blocker as measured with place-preference and self-stimulation behavior. Neuroscience.

[35] Kruppa J.A., Gossen A., Weiss. E.O., Kohls G., Grossheinrich N., Cholemkery H., Freitag C.M., Karges W., Wolfle E., Sinzig J. (2019). Neural modulation of social reinforcement learning by intranasal oxytocin in male adults with high-functioning autism spectrum disorder: A randomized trial. Neuropsychopharmacology.

[36] Grund T., Tang Y., Benusiglio D., Althammer F., Probst S., Oppenlander L., Neumann I.D., Grinevich V. (2019). Chemogenetic activation of oxytocin neurons: Temporal dynamics, hormonal release, and behavioral consequences. Psychoneuroendocrinology.

[37] Hurlemann R., Patin A., Onur O.A., Cohen M.X., Baumgartner T., Metzler S., Dziobek I., Gallinat J., Wagner M., Maier W. (2010). Oxytocin enhances amygdala-dependent, socially reinforced learning and emotional empathy in humans. J. Neurosci..

[38] Gamer M., Zurowski B., Buchel C. (2010). Different amygdala subregions mediate valence-related and attentional effects of oxytocin in humans. Proc. Natl. Acad. Sci. USA.

[39] Borland J.M., Grantham K.N., Aiani L.M., Frantz K.J., Albers H.E. (2018). Role of oxytocin in the ventral tegmental area in social reinforcement. Psychoneuroendocrinology.

[40] Dolen G., Darvishzadeh A., Huang K.W., Malenka R.C. (2013). Social reward requires coordinated activity of nucleus accumbens oxytocin and serotonin. Nature.

[41] Kent K., Arientyl V., Khachatryan M.M., Wood R.I. (2013). Oxytocin induces a conditioned social preference in female mice. J. Neuroendocrinol..

[42] Liberzon I., Trujillo K.A., Akil H., Young E.A. (1997). Motivational properties of oxytocin in the conditioned place preference paradigm. Neuropsychopharmacology.

[43] Song Z.M., Borland J.M., Larkin T.E., O’Malley M., Albers H.E. (2016). Activation of oxytocin receptors, but not arginine-vasopressin V1a receptors, in the ventral tegmental area of male Syrian hamsters is essential for the reward-like properties of social interactions. Psychoneuroendocrinology.

[44] Hung L.W., Neuner S., Polepalli J.S., Beier K.T., Wright M., Walsh J.J., Lewis E.M., Luo L., Deisseroth K., Dolen G. (2017). Gating of social reward by oxytocin in the ventral tegmental area. Science.

[45] Kosaki Y., Watanabe S. (2016). Conditioned social preference, but not place preference, produced by intranasal oxytocin in female mice. Behav. Neurosci..

[46] Leong K.C., Cox S., King C., Becker H., Reichel C.M. (2018). Oxytocin and Rodent Models of Addiction. Int. Rev. Neurobiol..

[47] Huston J.P., Silva M.A., Topic B., Muller C.P. (2013). What’s conditioned in conditioned place preference?. Trends Pharmacol. Sci..

[48] Adcock R.A., Thangavel A., Whitfield-Gabrieli S., Knutson B., Gabrieli J.D. (2006). Reward-motivated learning: Mesolimbic activation precedes memory formation. Neuron.

[49] Ikemoto S., Panksepp J. (1999). The role of nucleus accumbens dopamine in motivated behavior: A unifying interpretation with special reference to reward-seeking. Brain Res. Brain Res. Rev..

[50] Paval D., Miclutia I.V. (2021). The Dopamine Hypothesis of Autism Spectrum Disorder Revisited: Current Status and Future Prospects. Dev. Neurosci..

[51] Chevallier C., Kohls G., Troiani V., Brodkin E.S., Schultz R.T. (2012). The social motivation theory of autism. Trends Cogn. Sci..

[52] Damiano C.R., Aloi J., Dunlap K., Burrus C.J., Mosner M.G., Kozink R.V., McLaurin R.E., Mullette-Gillman O.A., Carter R.M., Huettel S.A. (2014). Association between the oxytocin receptor (OXTR) gene and mesolimbic responses to rewards. Mol. Autism.

[53] Love T.M. (2013). Oxytocin, motivation and the role of dopamine. Pharmacol. Biochem. Behav..

[54] Love T.M., Enoch M.A., Hodgkinson C.A., Pecina M., Mickey B., Koeppe R.A., Stohler C.S., Goldman D., Zubieta J.K. (2012). Oxytocin gene polymorphisms influence human dopaminergic function in a sex-dependent manner. Biol. Psychiatry.

[55] Rosenfeld A.J., Lieberman J.A., Jarskog L.F. (2011). Oxytocin, dopamine, and the amygdala: A neurofunctional model of social cognitive deficits in schizophrenia. Schizophr. Bull..

[56] Huber D., Veinante P., Stoop R. (2005). Vasopressin and oxytocin excite distinct neuronal populations in the central amygdala. Science.

[57] Romero-Fernandez W., Borroto-Escuela D.O., Agnati L.F., Fuxe K. (2013). Evidence for the existence of dopamine D2-oxytocin receptor heteromers in the ventral and dorsal striatum with facilitatory receptor-receptor interactions. Mol. Psychiatry.

[58] Peris J., MacFadyen K., Smith J.A., de Kloet A.D., Wang L., Krause E.G. (2017). Oxytocin receptors are expressed on dopamine and glutamate neurons in the mouse ventral tegmental area that project to nucleus accumbens and other mesolimbic targets. J. Comp. Neurol..

[59] de la Mora M.P., Perez-Carrera D., Crespo-Ramirez M., Tarakanov A., Fuxe K., Borroto-Escuela D.O. (2016). Signaling in dopamine D2 receptor-oxytocin receptor heterocomplexes and its relevance for the anxiolytic effects of dopamine and oxytocin interactions in the amygdala of the rat. Biochim. Biophys. Acta.

[60] Fuxe K., Borroto-Escuela D.O., Romero-Fernandez W., Ciruela F., Manger P., Leo G., Diaz-Cabiale Z., Agnati L.F. (2012). On the role of volume transmission and receptor-receptor interactions in social behaviour: Focus on central catecholamine and oxytocin neurons. Brain Res..

[61] Young L.J., Lim M.M., Gingrich B., Insel T.R. (2001). Cellular mechanisms of social attachment. Horm. Behav..

[62] Mu P., Yu L.C. (2007). Valproic acid sodium inhibits the morphine-induced conditioned place preference in the central nervous system of rats. Neurosci. Lett..

[63] Spyraki C., Kazandjian A., Varonos D. (1985). Diazepam-induced place preference conditioning: Appetitive and antiaversive properties. Psychopharmacology.

[64] Miller C.K., Halbing A.A., Patisaul H.B., Meitzen J. (2021). Interactions of the estrous cycle, novelty, and light on female and male rat open field locomotor and anxiety-related behaviors. Physiol. Behav..

[65] Freitag C.M., Jensen K., Elsuni L., Sachse M., Herpertz-Dahlmann B., Schulte-Ruther M., Hanig S., von Gontard A., Poustka L., Schad-Hansjosten T. (2016). Group-based cognitive behavioural psychotherapy for children and adolescents with ASD: The randomized, multicentre, controlled SOSTA-net trial. J. Child. Psychol. Psychiatry.

[66] Andari E., Duhamel J.R., Zalla T., Herbrecht E., Leboyer M., Sirigu A. (2010). Promoting social behavior with oxytocin in high-functioning autism spectrum disorders. Proc. Natl. Acad. Sci. USA.

[67] Lefter R., Ciobica A., Antioch I., Ababei D.C., Hritcu L., Luca A.C. (2020). Oxytocin Differentiated Effects According to the Administration Route in a Prenatal Valproic Acid-Induced Rat Model of Autism. Medicina.

[68] Wang Y., Zhao S., Liu X., Zheng Y., Li L., Meng S. (2018). Oxytocin improves animal behaviors and ameliorates oxidative stress and inflammation in autistic mice. Biomed. Pharmacother..

